# CSF metabolomics alterations after aneurysmal subarachnoid hemorrhage: what do we know?

**DOI:** 10.1007/s13760-023-02266-2

**Published:** 2023-04-30

**Authors:** Wing Mann Ho, Franziska A. Schmidt, Claudius Thomé, Ondra Petr

**Affiliations:** Department of Neurosurgery, Medical University Innsbruck, Anichstrasse 35, 6020 Innsbruck, Austria

**Keywords:** Subarachnoid hemorrhage, Metabolomics, Cerebrospinal fluid, Biomarker, Delayed cerebral ischemia, Amino acids

## Abstract

**Purpose:**

The purpose of this mini review is to describe metabolomics in cerebrospinal fluid (CSF) and its potential in aneurysmal subarachnoid hemorrhage (aSAH). In brain injury, patients’ micro dialysis enables detecting biochemical change in brain tissue. Indicators for ischemia were detected such as lactate, pyruvate, glucose, and glutamate. In aSAH patients, the pathophysiology and the factor for poor outcome are not completely understood yet. Routine use of biomarkers in CSF, particularly in aSAH patients, is still lacking.

**Methods:**

This mini review was performed on the role of metabolomics alterations after aneurysmal subarachnoid hemorrhage.

**Results:**

We identified five clinical studies that addressed metabolomics in patients with aneurysmal subarachnoid hemorrhage.

**Conclusion:**

There is increasing evidence suggesting that biomarkers can give insight in the pathogenesis and can serve as an outcome predictor. In this mini review, we present a brief overview of metabolomics profiling in neuroscience and wish to discuss the predictive and therapeutic value in aSAH patients.

**Supplementary Information:**

The online version contains supplementary material available at 10.1007/s13760-023-02266-2.

## Introduction

Regardless of the extensive experimental and clinical research efforts, aneurysmal subarachnoid hemorrhage (aSAH) remains a devastating disease, frequently striking patients in a fairly young age without any warning signs. Half of patients with aSAH are younger than 55 years old. Most survivors remain significantly impaired in their daily lives [1]. Post-hemorrhagic pathophysiological mechanisms are multifactorial and complex and factors predicting poor outcome or delayed cerebral ischemia (DCI) are not completely understood. Thus, the incorporation of innovative research strategies is pivotal. The advent of metabolomic approaches allows the simultaneous, large-scale screening of numerous metabolites in a biological sample. A major benefit of metabolomic research is novel metabolites that may be discovered and associated with a disease process for the first time, without the demand for an existing hypothesis. Despite considerable success in brain research, to date, metabolomics has hardly been applied for studying SAH-related changes in CSF [3, 4].

In this mini review, we present a brief overview of CSF metabolomics profiling in neuroscience and discuss its potential predictive and therapeutic value in aSAH patients.

## Cerebrospinal fluid metabolome

Apart from numerous vital functions of CSF such as hydromechanical cerebral protection and salubrious homeostasis of the brain, specific CSF conditions may mirror pertinent alterations offering an in-depth insight into the cerebral metabolite production rates and its disorders for instance aSAH. Metabolomics methods have identified appreciable number of biomarkers for cerebral diseases including their progression [3, 5–7].

Metabolites are small molecules (< 1500 Da) in various body fluids which can influence cell response locally and systemically, possibly reflecting a disease progression [8]. Metabolites can be activated under specific conditions or can change during a disease progress or depending on drug interactions [4, 9]. The term “metabolome” is derived from the term metabolism and refers to a set of molecules within a biological sample (e.g., cerebrospinal fluid) [10]. In other words, metabolomics describes the systematic analysis of metabolites [11, 12].

In 2008, a very first systematic analysis of the human cerebrospinal fluid metabolome demonstrated that 41–70 CSF compounds can be detected with nuclear magnetic resonance spectroscopy (NMR), gas chromatography–mass spectrometry (GC–MS) or Fourier transform mass spectrometry coupled with liquid chromatography (LC–FTMS). The authors of the study also described that CSF is a metabolically diverse fluid with 33 different compound categories. Of note, CSF is rich in amino acids, sugars, and inorganic salts [5].

Nowadays, metabolomic approaches are used in several neurological diseases to particularize diagnosis, prognosis, and monitor treatments. Metabolomics has been applied in many degenerative disorders. For instance, various metabolites are being routinely used in diagnostics and treatment for Alzheimer´s disease, amyotrophic lateral sclerosis, epilepsy, Parkinson’s disease, multiple sclerosis, and stroke [11].

## CSF metabolomics in aneurysmal subarachnoid hemorrhage

In respect of aSAH, established or clinically verified biomarkers are still lacking or are not yet used routinely in clinical practice. Discovery of novel specific indicators for, i.e., DCI and poor clinical outcome is urgently needed. However, to our best knowledge, there are only three recent studies dealing with metabolomics profiling of CSF after aSAH.

In a 2017 European study, Sokół et al. revealed that in their liquid chromatography–mass spectrometry/mass spectrometry (LC–MS/MS) of 49 CSF samples from 23 patients analyzing 33 amino acids and related compounds, 27 of them were significantly elevated within the first 3 days after aSAH. The investigation showed that the patterns differ between poor and good outcome. In particular, during early brain injury (EBI) after aSAH, concentration of nine amino acids was significantly higher in patients with poor outcome compared to those with good outcome: taurine, aspartic acid, citrulline, glutamic acid, gamma-amino-butyric acid, 3-methyl-histidine, ornithine, cystathionine, and isoleucine. On day 5 post-SAH, glutamic acid was the only amino acid that significantly increased. Interestingly, higher levels of excitatory amino acids (i.e., glutamic acid, aspartic acid, and 2-amino-adipic acid) appear to predict a poor outcome [13]. Similar findings were described in an American study in the same year. Of 97 metabolites identified with known chemical structures, 16 metabolites, primarily free amino acids, significantly changed their concentration with time. Of these, six metabolites (i.e., 2-hydroxyglutarate, tryptophan, glycine, proline, isoleucine, and alanine) strongly correlated with Glasgow Outcome Score (GOS) of patients at 1-year post-SAH [14]. Importantly, none of these metabolites correlated with vasospasm.

The first prospective study analyzed endogenous metabolites in patients with ruptured intracranial aneurysms and aSAH Fisher Grade III and IV compared to patients with elective aneurysms. CSF samples were consecutively collected before aneurysm treatment (the time point of EVD placement), intraoperatively, 6 h later, and daily thereafter for 10 days. The study showed that amino acids, biogenic amines, and acylcarnitine levels continuously increased over the time starting as early as 6 h after aSAH onset. Taurine concentration, however, was initially elevated directly after onset and started decreasing after only 6 h. Other analyzed metabolites such as hydroxysphingomyelins, lysophosphatidylcholines, and sphingomyelins showed temporarily increased levels immediately after aSAH with an ensuing decrease to CSF concentrations within the first 6 h that are comparable with the non-hemorrhagic CSF. The study presented significant consecutive alterations of various metabolites in the longitudinal course of aSAH. There was a peak of structural amino acids as early as within the first 6 h after aneurysm treatment. However, the authors concluded that further evaluation of CSF metabolites and compounds and their time-dependent alterations may elucidate pathophysiological processes after aSAH, potentially providing new predictive biomarkers related to aSAH-specific parameters and outcomes [16].

A Chinese study published in 2018 showed data of 40 subarachnoid hemorrhage patients and the control group of 6 patients. Neurological outcome was evaluated 12 months after discharge. Interestingly, the group showed that there is no significant variation among CSF metabolome in subarachnoid hemorrhage patients with different fisher scales (amount and distribution of blood). However, sub-analysis showed that pyruvate metabolism was altered in subarachnoid hemorrhage patients, especially in those with a high Hunt–Hess scale (clinical condition). Pyruvate level is associated with WFNS (focal motor deficit) grading scale above III. In retrospect, patients with unfavorable outcome had significantly altered amino acid metabolism and lipid biosynthesis [17].

The work of an American group which was published in 2020 aimed to identify the leading CSF metabolites associated with poor outcome, as determined by the modified Rankin Scale (mRS) at discharge and at 90 days after discharge. The authors used 81 CSF samples. Through orthogonal partial least squares-discriminant analysis, symmetric dimethylarginine (SDMA), dimethylguanidine valeric acid (DMGV), and ornithine were detected as the markers associated with poor outcome [18].

All five aforementioned clinical studies dealing with CSF metabolomics after aSAH provide a first very interesting finding of various metabolites that can be instrumental in further innovative research strategies.

## Discussion

In summary, metabolomics of post-hemorrhagic CSF provides a unique signature of multifactorial pathophysiologic processes in the brain after aSAH. A published study in 2005 described metabolomics as a systematic study of unique chemical fingerprints that specific cellular processes leave behind that provides dynamic information [15]. While still in its infancy, we shall perceive the promising potential and possible future application of CSF metabolomics post-aSAH for clinical practice. All, Ho et al., Sokół et al., Lu et al., Koch et al., and Li et al. showed, in their clinical observational studies, several metabolites linked to aSAH and its outcomes. All authors concurred in significantly elevated levels of several amino acids in post-aSAH CSF time courses. So far, only these five clinical studies with a small number of patients are available in the literature (Supplementary Table 1). It can be discussed that the studies might have been underpowered. In our opinion, further thorough systematic evaluation of all identified CSF metabolites and compounds alongside with their time-dependent longitudinal alterations may be very helpful to possibly clarify pathophysiological processes after aSAH, with a potential of discovering new clinically relevant biomarkers related to aSAH-specific events.

**Fig. 1:**
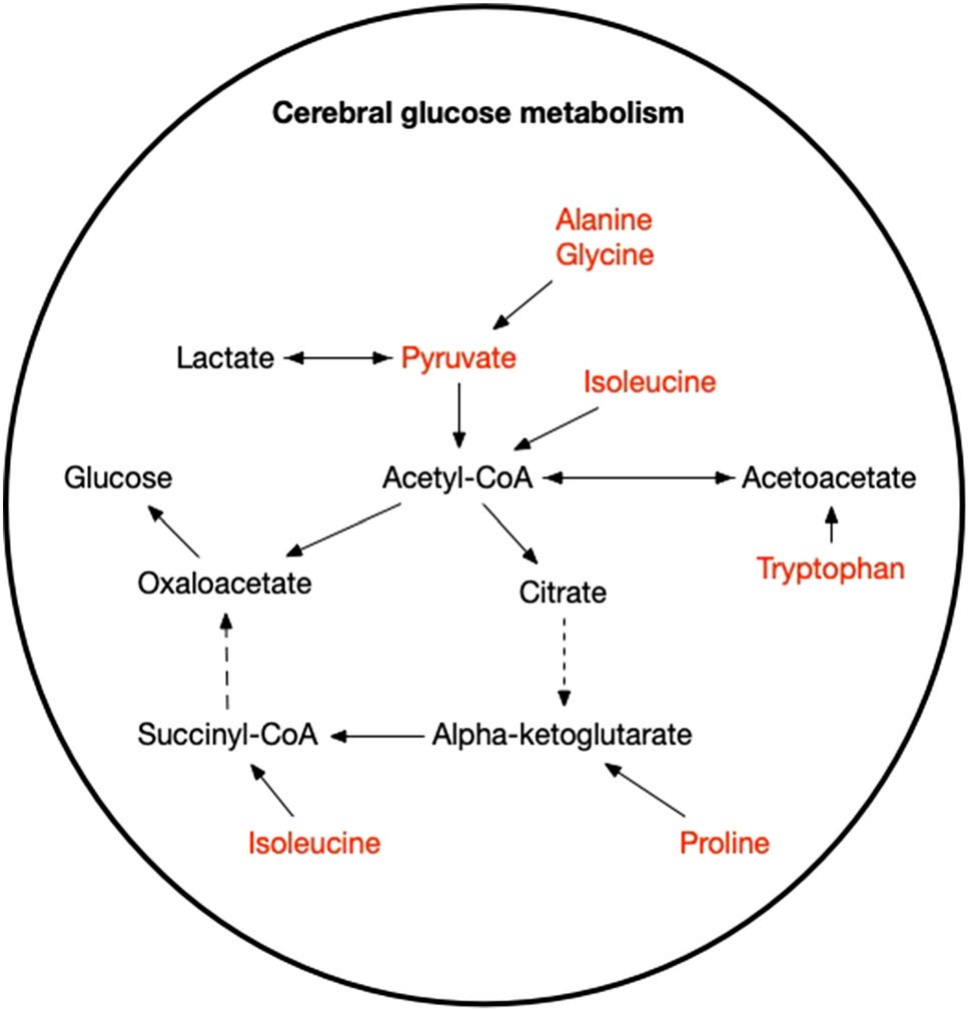
Overview of the cerebral glucose metabolism known as the citrate cycle and red marked are the significantly altered metabolites after aSAH [14, 16, 17]

**Fig. 2:**
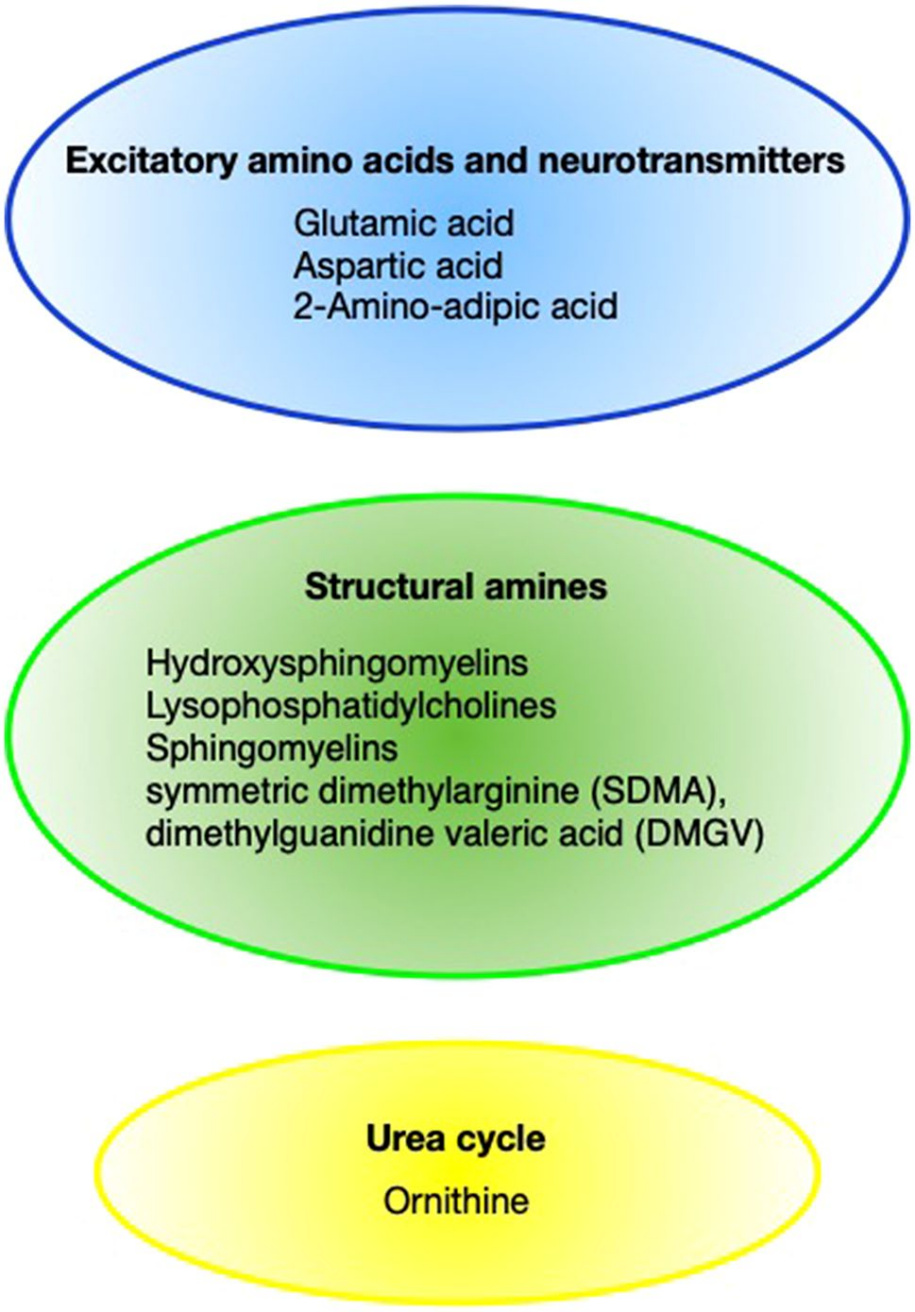
Significantly altered metabolites after aSAH and their role in physiological processes [13, 16, 18]

Several significant altered compounds in those studies are found in the cerebral glucose metabolism (Fig. 1). Further, metabolites as excitatory amino acids in the neurotransmitter formation and structural amine levels were changed after aSAH (Fig. 2).

Future metabolomics profiling in both untargeted and targeted ways in aSAH patients should allow acquiring further insights into the global cerebral endogenous metabolism after aSAH with in-depth monitoring of pertinent metabolomic changes, and therefore potential identification of aSAH-specific biomarkers.

### Supplementary Information

Below is the link to the electronic supplementary material.

Supplementary file1 (DOCX 110 KB)

## Data Availability

Availability of Data and Material not applicable.

## Abbreviations

CSF: Cerebrospinal fluid
SAH: Subarachnoid hemorrhage
aSAH: Aneurysmal subarachnoid hemorrhage
GABA: Gamma-aminobutyric acid
DCI: Delayed cerebral ischemia
NMR: Nuclear magnetic resonance
GC–MS: Gas chromatography–mass spectrometry
LC–FTMS: Fourier transform mass spectrometry coupled with liquid chromatography
EBI: Early brain injury
GOS: Glasgow Outcome Score
