# Supplementary material for: CSF metabolomics alterations after aneurysmal subarachnoid hemorrhage: what do we know?
Source: Acta Neurol Belg. 2023 Apr 30;123(6):2111–4. doi: 10.1007/s13760-023-02266-2 (PMC10682053; doi:10.1007/s13760-023-02266-2)
Supplement: Supplementary file 1 — Supplementary file1 (DOCX 110 KB) [file 13760_2023_2266_MOESM1_ESM.docx]

**Table**

**Table 1:** Summary of the studies dealing with CSF metabolomics in aSAH.
